# *In vitro* antibacterial effect of *Withania somnifera* root extract on *Escherichia coli*

**DOI:** 10.14202/vetworld.2015.57-60

**Published:** 2015-01-17

**Authors:** Mamta Kumari, R. P. Gupta

**Affiliations:** Department of Veterinary Pathology, Lala Lajpat Rai University of Veterinary and Animal Sciences, Hisar, Haryana, India

**Keywords:** antibacterial, Ashwagandha, *Withania somnifera*, root extract

## Abstract

**Aim:**

The aim was to investigate antibacterial activity of *Withania somnifera* (Ashwagandha), an Indian traditional medicinal plant against *Escherichia coli* O78, a pathogenic strain.

**Materials and Methods:**

Two-fold serial dilutions of 20% aqueous *W. somnifera* root (WSR) extract were inoculated with *E. coli* O78 @ 1*10^7^ colony forming units grown in nutrient broth. Following inoculation, turbidity optical density was measured by spectrophotometer at 600 nm in all the tubes at 0, 2, 4, 6 and 8 h of incubation at 37°C.

**Result:**

The results revealed that the maximum inhibition of bacterial growth was observed at 1:8 dilution of WSR extract. The highest dilution of the extract that showed inhibited growth of the test organism when compared with control was 1:16. Therefore, the minimum inhibitory concentration of aqueous extract of WSR is 1:16.

**Conclusion:**

It is concluded that WSR possessed good antibacterial activity, confirming the great potential of bioactive compounds and its rationalizing use in health care.

## Introduction

In the last few years, there has been an exponential growth in the field of herbal medicine, and these drugs are gaining popularity both in developing and developed countries because of their natural origin and less side-effects. *Withania somnifera* is an important medicinal plant, a small, woody shrub 60-200 cm high, in the Solanaceae family, which is described under many common names such as Ginseng and Ashwagandha. Withanolides are the major active constituents of *W. somnifera*, which are isolated from its root and leaves. *Withaferin A* and *Withanolide D* are the two main *Withanolides* that contribute to most of the biological activity of *W. somnifera* [1]. The total alkaloid content of Indian root varies from 0.13% to 0.31%. *W. somnifera* has been used as an anti-oxidant, adaptogen, aphrodisiac, liver tonic, anti-inflammatory agent, astringent and antibacterial agent [2,3]. Several reports have demonstrated the immunomodulator and anti-tumour activity of root extract of *W. somnifera* [4-6].

The use of antibiotics has led to success in limiting most of the prevalent bacterial diseases which affected man and animals in epidemic proportions. At the same time, inadvertent and overuse of antibiotics resulted into emergence of resistance in an organism against the commonly used antibiotics. In domestic poultry, avian colibacillosis is frequently associated with *Escherichia coli* strains of serotypes O78:K80, O1:K1 and O2:K1. These *E. coli* strains are often resistant to a large number of antimicrobials such as cephradine, tetracyclines, chloramphenicol, sulfonamides, β-lactam antibiotics, amino-glycosides and fluoroquinolones [7,8]. The emergence of drug-resistant organisms necessitates the search for an alternative source of antimicrobial agents that has necessitated a study of the effect of herbal plants against *E. coli*. WHO has recommended development and use of environment-friendly alternative methods to control diseases in poultry and other food producing animals [9]. Indian traditional system of medicine, i.e., Ayurveda has successfully employed plant derived products in the treatment of almost all types of ailments in human and animals.

The present work was carried out to study the *in vitro* antibacterial activity of root extracts of *W. somnifera* against *E. coli* O78.

## Materials and Methods

### Preparation of extracts from *W. somnifera* roots (WSR)

WSR was collected from Medicinal and Aromatic Plants Section, Department of Genetics and Plant Breeding, College of Agriculture, CCSHAU, Hisar. Roots of Ashwagandha were washed and then dried in the shade and then powdered. For aqueous extraction, 20 g of root powder was dissolved in 100 ml of distilled water and boiled at 100°C for 6 h. Then it was filtered through Whatmann (No. 1) filter paper and stored at 4°C till further use [10].

### Determination of minimum inhibitory concentration (MIC)

The MIC was defined as the lowest concentration of the compound to inhibit the growth of microorganisms. The minimum inhibitory concentration values were determined by broth dilution assay and colony-forming units (CFU). The test organism (*E. coli* O78), isolated from natural case of *E. coli* infection was obtained from Department of Veterinary Epidemiology and Public Health.

### Broth dilution assay

Two-fold serial dilutions of 20% aqueous WSR extract were prepared in sterilized test tubes with sterile normal saline solution (NSS) up to 1:32 dilution. Based on the dilution, there were eight groups. Group A represented undiluted extract and groups B to F represented diluted extract from1:2 to 1:32 dilution, respectively. Group G contained only NSS without the root extract, representing control positive, whereas Group H contained NSS and (20%) root extract and is kept uninoculated representing control negative. These tubes were taken in triplicates. The total amount of each dilution was kept 2 ml. Equal volume of double strength (×2) of nutrient broth was added in tubes of various groups so as to make normal concentration of nutrients after the addition of medium.

All the groups except control negative group were inoculated with 0.1 ml of broth culture of *E. coli* O78 containing 1 × 10^7^ CFU. Following inoculation all the tubes were kept at 37°C and optical density (OD) was measured with spectrophotometer at 600 nm at 0, 2,4,6 and 8 h of incubation for bacterial growth (turbidity). During the measurement of OD in the groups containing WSR extract, the blank was set with their respective control negative tubes lacking *E. coli* to rule out the absorbance due to color of WSR extract.

The highest dilution of plant extract that showed inhibited growth of the test organism as compared with control was considered as MIC [11]. The highest dilution of the plant extract which killed all the test organisms was considered as minimum bactericidal or lethal concentration (MBC).

### Determination of CFU

A serial 10 fold dilutions of the above-mentioned groups was made after 8 h of incubation in NSS. 100 µl from each of the diluted test tubes was spread evenly on MacConkey’s Lactose agar with the help of sterile spreader. The plates were incubated at 37°C overnight, and the number of colonies was counted.

Percent inhibition of bacterial growth in various dilutions of root extract with respect to growth in control positive was calculated using the formula [12]: (CFU in control positive-CFU in test group/CFU in control positive) × 100

### Statistical analysis

The data were subjected to statistical analysis by applying two-way ANOVA using Statistical Package for Social Sciences 17th version, IBM, USA. Difference between means tested using Tukey (HSD) *Post hoc* comparisons and significance was set at p<0.05.

## Results

### Broth dilution assay

Mean values of optical density indicating the turbidity due to bacterial growth in various dilutions of WSR extract at different incubation periods is shown in Table 1. There was no growth in control negative tubes (Group H). There was a statistically significant interaction between the OD values of various dilutions of the extract at various time intervals of incubation. Group A (undiluted extract) revealed higher bacterial growth when compared to control at almost every time interval. In Groups B (1:2) and C (1:4), the turbidity in tubes was equal to that in control positive Group G up to 4 h, but at 6 h of incubation there was significantly lower bacterial growth in Groups B and C. Significant changes in bacterial growth were observed in Group D (1:8 dilution). The mean OD value corresponding to bacterial growth was significantly lower in Group D when compared to Group G from 2 h onwards. The lowest turbidity was observed at 4 hours of incubation in Group D. Similarly, turbidity due to bacterial growth was lower in Group E when compared to control, but higher compared with Group D at 4 and 6 h of incubation. In Group F, there was increase in turbidity due to growth of bacteria and the mean OD values did not differed significantly with control Group G. The analysis of results showed that the highest dilution of plant extract that showed inhibited growth of test organism as compared with control was 1:16 (Group E). Therefore, the MIC of 20% aqueous extract of WSR was 1:16. However, maximum inhibition of bacterial growth was observed in Group D at 1:8 dilution (i.e. at 2.5% WSR extract). None of the extract containing tubes revealed complete inhibition of bacterial growth (i.e. no growth) with any turbidity and so minimum bactericidal/lethal concentration could not be observed.

**Table-1:** Mean OD values of turbidity due to bacterial growth (*E. coli*) at various dilutions of 20% aqueous WSR extract

20% Aqueous WSR extract dilutions	Incubation time					Overall mean

	0 h	2 h	4 h	6 h	8 h	
Group A (Undiluted)	0.49^e^±0.00	1.49^d^±0.001	1.38^e^±0.012	1.10^b^±0.003	1.12^d^±0.039	1.11^e^±0.013
Group B (1:2)	0.45^d^±0.001	1.46^cd^±0.00	1.29^d^±0.011	0.76^a^±0.012	0.81^c^±0.007	0.95^d^±0.013
Group C (1:4)	0.45^d^±0.00	1.44^c^±0.002	1.24^d^±0.006	0.62^a^±0.01	0.65^b^±0.007	0.88^c^±0.013
Group D (1:8)	0.42^c^±0.012	0.42^a^±0.003	0.27^a^±0.001	0.74^a^±0.003	0.75^c^±0.003	0.52^a^±0.013
Group E (1:16)	0.40^b^±0.001	0.43^a^±0.001	0.62^b^±0.01	1.13^b^±0.136	0.55^a^±0.007	0.63^b^±0.013
Group F (1:32)	0.34^a^±0.001	1.37^b^±0.03	1.13^c^±0.044	1.36^bc^±0.063	0.53^a^±0.007	0.95^d^±0.013
Group G (Control positive)	0.34^a^±0.001	1.46^cd^±0.001	1.22^d^±0.009	1.42^c^±0.019	0.54^a^±0.010	1.00^d^±0.013
Group H (Control negative)	0.00±0.00	0.00±0.00	0.00±0.00	0.00±0.00	0.00±0.00	0.00±0.00

Mean±SE with unlike superscript in the columns differ significantly (p<0.05), SE=Standard error, WSR=*Withania somnifera* root, OD: Optical density, *E. coli=Escherichia coli*

### CFU

CFU in different groups after 8 hours of incubation are given in Table-2. Group H (control negative) revealed no bacterial colony. Groups A-E revealed significantly lower CFU as compared to Group G. However, the lowest count was observed in Group D (1:8) as compared to all other groups. Group E (1:16) revealed significantly lower CFU as compared to Group F (1:32) and Group G (control positive) but higher count as compared to Group D. Therefore, least growth of bacteria was observed in Group D and Group E was minimum inhibitory group.

**Table-2:** Total CFU of *E. coli* in different dilutions of WSR extract

20% Aqueous WSR extract dilutions	CFU/100 µl after 24 h	Percent inhibition w.r.t. control positive
Group A (Undiluted)	9.40^b^±3.05×10^6^	38.68
Group B (1:2)	10.33^bc^±5.21×10^6^	32.62
Group C (1:4)	12.27^cd^±4.06×10^6^	19.96
Group D (1:8)	6.53^a^±2.91×10^6^	57.40
Group E (1:16)	8.4^ab^±2.31×10^6^	45.21
Group F (1:32)	13.60^de^±2.31×10^6^	11.29
Group G (control positive)	15.33^e^±8.11×10^6^	0
Group H (control negative)	-	-

Mean±SE with unlike superscript in the column differ significantly (p<0.05), SE=Standard error, *E. coli=Escherichia coli*, WSR=*Withania somnifera* root, CFU=Colony forming units

Percent inhibition of Bacterial Growth: All the extract containing groups revealed inhibition of bacterial growth with respect to control positive. However, maximum percent inhibition (57. 40%) of bacterial growth was observed in Group D (1:8) whereas minimum inhibition was observed in Group F (11.29%) w.r.t. to control positive.

## Discussion

The results revealed that WSR possessed good antibacterial activity, confirming the great potential of bioactive compounds present in the roots of this plant in health care. Although the bactericidal/lethal effect of the extract was not observed in any of the dilutions and even in the undiluted solution, but its growth inhibitory effect against *E. coli* was revealed. Minimum turbidity was observed at 1:8 dilution (Group D) and its corresponding CFU count was also lowest amongst all the groups. The percent inhibition of growth was maximum in Group D. Group F revealed higher turbidity and colony forming units as compared to Group E which might be due to absence of minimum inhibitory concentration of extract in these tubes to inhibit the growth of bacteria. The antimicrobial effect of any substance is observed only when a certain specific concentration of that substance is reached to inhibit the bacteria and below this concentration bacterial growth is not inhibited. This study revealed that 1:16 dilution of a 20% aqueous extract of WSR is the minimum inhibitory concentration against *E. coli* bacteria. Antibacterial activity of leaves and roots of *W. somnifera* extract against *E.coli* has been observed *in vitro* by disc diffusion assay and *in vivo* in Guinea pigs [13,14]. A study revealed that the aqueous extract of WSR inhibited the growth of bacteria in dose-dependent manner [15]. Similarly, *in vitro* antibacterial activity of *W. somnifera* against Gram-negative bacteria, particularly *Salmonella typhi* and *E. coli* has been observed by other workers [16-18].

## Conclusion

It is concluded that the aqueous root extract of *W. somnifera* hold an excellent potential as an antibacterial agent against *E. coli* O78 and ascertains the value of medicinal plants used in Ayurveda, which could help in the development of an alternative drug.

## Authors’ Contributions

MK conducted the present study, and RPG helped in interpretation of results and data. Both the authors contributed in designing the present study and preparation of the manuscript. Both authors read and approved the final manuscript.
